# Constant rate infusion of diazepam or propofol for the management of canine cluster seizures or status epilepticus

**DOI:** 10.3389/fvets.2022.1005948

**Published:** 2022-11-17

**Authors:** Giulia Cagnotti, Sara Ferrini, Giorgia Di Muro, Giuliano Borriello, Cristiano Corona, Luca Manassero, Eleonora Avilii, Claudio Bellino, Antonio D'Angelo

**Affiliations:** ^1^Department of Veterinary Science, University of Turin, Torino, Italy; ^2^Istituto Zooprofilattico del Piemonte, Liguria e Valle d'Aosta, Torino, Italy

**Keywords:** dog, neurology, epilepsy, cluster seizures, status epilepticus, diazepam, propofol

## Abstract

**Introduction:**

Cluster seizures (CS) and status epilepticus (SE) in dogs are severe neurological emergencies that require immediate treatment. Practical guidelines call for constant rate infusion (CRI) of benzodiazepines or propofol (PPF) in patients with seizures not responding to first-line treatment, but to date only few studies have investigated the use of CRI in dogs with epilepsy.

**Study design:**

Retrospective clinical study.

**Methods:**

Dogs that received CRI of diazepam (DZP) or PPF for antiepileptic treatment during hospitalization at the Veterinary Teaching Hospital of the University of Turin for CS or SE between September 2016 and December 2019 were eligible for inclusion. Favorable outcome was defined as cessation of clinically visible seizure activity within few minutes from the initiation of the CRI, no seizure recurrence within 24 h after discontinuation of CRI through to hospital discharge, and clinical recovery. Poor outcome was defined as recurrence of seizure activity despite treatment or death in hospital because of recurrent seizures, catastrophic consequences of prolonged seizures or no return to an acceptable neurological and clinical baseline, despite apparent control of seizure activity. Comparisons between the number of patients with favorable outcome and those with poor outcome in relation to type of CRI, seizure etiology, reason for presentation (CS or SE), sex, previous AED therapy and dose of PPF CRI were carried out.

**Results:**

A total of 37 dogs, with 50 instances of hospitalization and CRI administered for CS or SE were included in the study. CRI of diazepam (DZP) or PPF was administered in 29/50 (58%) and in 21/50 (42%) instances of hospitalization, respectively. Idiopathic epilepsy was diagnosed in 21/37 (57%), (13/21 tier I and 8/21 tier II); structural epilepsy was diagnosed in 6/37 (16%) of which 4/6 confirmed and 2/6 suspected. A metabolic or toxic cause of seizure activity was recorded in 7/37 (19%). A total of 38/50 (76%) hospitalizations were noted for CS and 12/50 (24%) for SE. In 30/50 (60%) instances of hospitalization, the patient responded well to CRI with cessation of seizure activity, no recurrence in the 24 h after discontinuation of CRI through to hospital discharge, whereas a poor outcome was recorded for 20/50 (40%) cases (DZP CRI in 12/50 and PPF CRI in 8/50). Comparison between the number of patients with favorable outcome and those with poor outcome in relation to type of CRI, seizure etiology, reason for presentation (CS or SE), sex and previous AED therapy was carried out but no statistically significant differences were found.

**Conclusions:**

The present study is the first to document administration of CRI of DZP or PPF in a large sample of dogs with epilepsy. The medications appeared to be tolerated without major side effects and helped control seizure activity in most patients regardless of seizure etiology. Further studies are needed to evaluate the effects of CRI duration on outcome and complications.

## Introduction

Cluster seizures (CS) and status epilepticus (SE) in dogs are severe neurological emergencies; the case fatality rate ranges between 25.3 and 28.5% (1–3). Though such medical emergencies require immediate treatment, the optimal therapeutic approach has yet to be defined (4). Proposed treatment guidelines call for constant rate infusion (CRI) of benzodiazepines or propofol (PPF) in patients with seizures not responding to first-line benzodiazepines and non-anesthetic drugs (5, 6). Few studies to date have investigated the use of CRI in dogs with epilepsy (1, 7). With this retrospective study we wanted to describe the use of two types of CRI in dogs with refractory seizure activity presenting to a single veterinary teaching hospital.

## Materials and methods

### Study population

The study sample was dogs presenting with convulsive CS or SE to the Veterinary Teaching Hospital (VTH), Department of Veterinary Science, University of Turin. The dogs that received CRI of diazepam (DZP) or PPF for antiepileptic treatment during hospitalization between September 2016 and December 2019 were identified by medical record search. Inclusion criteria were SE or CS confirmed by owners' detailed description/video recording or evaluation at admission/during hospitalization, and primary management of the case by a board-certified neurologist (ADA) or a neurologist in training (GC) under the supervision of a board-certified neurologist. Patients were excluded if the emergency treatment protocol did not follow the standardized guidelines in place at our Institution or if data were missing for CRI (type of medication, dosage, duration, and outcome).

### Data collection and definitions

Data extracted from the medical records included: breed, weight, sex, neutering status, age at first seizure, age at hospitalization, history of seizures, previous antiepileptic drug (AED) administration (type and dosage), seizure etiology, reason for presentation (CS or SE), number of seizures (in patients with CS) or seizure duration before presentation (in patients with SE), emergency treatment protocol, type of AED for CRI, duration, and dosage, duration of hospitalization, and treatment outcome.

CS were clinically defined as the occurrence of two or more epileptic seizures within a 24-h period; SE was defined as seizure activity lasting more than 5 min or the occurrence of two or more epileptic seizures without complete recovery of consciousness in between them (8).

Seizure etiology was classified according to International Veterinary Epilepsy Task Force guidelines: reactive seizures, idiopathic epilepsy (tier I and II), and structural epilepsy (suspected or confirmed). Reactive seizures were defined according to history of possible or confirmed exposure to toxic agents or based on hematological laboratory test results (8). Dogs were classified as having idiopathic epilepsy if their first seizure occurred between 6 months and 6 years of age, two or more unprovoked seizures occurred >24 h apart, if the interictal neurological examination was normal (except for AED-induced neurologic abnormalities and post-ictal neurologic deficits), and if no clinically significant abnormalities were identified on minimum database blood tests and urinalysis (tier I) and unremarkable magnetic resonance imaging (MRI) of the brain and cerebrospinal fluid (CSF) analysis (tier II). Structural epilepsy was diagnosed when reactive causes of seizures were ruled out, along with signalment, history, an abnormal interictal neurological examination (suspected structural epilepsy) and anomalous MRI and CSF findings or necropsy (confirmed structural epilepsy). Dogs were classified as having undefined epilepsy if no investigations could be performed and no follow up was available.

Favorable outcome was defined as cessation of clinically visible seizure activity within few minutes from the initiation of the CRI, no seizure recurrence within 24 h after discontinuation of CRI through to hospital discharge, and clinical recovery. Poor outcome was defined as recurrence of seizure activity despite treatment or death in hospital (either by euthanasia or spontaneous) because of recurrent seizures, catastrophic consequences of prolonged seizures (such as cardiac arrhythmias, acute renal failure, rhabdomyolysis, hemorrhagic diarrhea, ab ingestis pneumonia) or no return to an acceptable neurological and clinical baseline, despite apparent control of seizure activity.

### Treatment protocol

At our Institution the emergency guidelines for dogs presenting with CS or SE call for administration of rectal/intravenous (IV) DZP (1–2 mg/kg if the patient is seizuring at presentation), followed by IV phenobarbital (4–5 mg/kg q8h) and rectal levetiracetam (LEV) (40 mg/kg one shot). CRI of DZP (0.5 mg/kg/h) or PPF (0.1–0.2 mg/kg/min) is initiated when there is persistence or rapid recurrence of seizure activity despite emergency treatment. The choice between the two AEDs is made by a board-certified neurologist (ADA) or a neurologist in training (GC) under the supervision of a board-certified neurologist for each individual case. In particular, DZP CRI is initiated when after an initial resolution of seizures with the emergency treatment protocol, these recur in a short period of time (within few hours) or when despite an initial improvement of convulsive activity, no complete resolution is obtained. A PPF CRI is considered when despite the emergency treatment protocol there's no modification of the seizure activity.

## Statistical analysis

Descriptive statistics and statistical analyses were performed using commercially available software (R version 4.1.3—November 2021). Continuous variables were tested for normality distribution using the Shapiro-Wilk test and were found to be non-parametric. Standard descriptive statistics are reported as median and interquartile range (IQR) for continuous variables and categorical variables as percentage and frequency. Comparisons between the number of patients with favorable outcome and those with poor outcome in relation to type of CRI, seizure etiology, reason for presentation (CS or SE) sex, previous AED therapy and dose of PPF CRI were carried out using Fisher's two-tailed exact test, chi-square test, or Wilcoxon ranked-sum two tailed test as appropriate. For comparisons between outcome and etiological groups, suspected and confirmed idiopathic epilepsy and suspected and confirmed structural epilepsy were combined/grouped together. Statistical significance was set at *P* < 0.05.

## Results

The study sample was 56 dogs, with 70 instances of hospitalization and CRI administered for CS or SE. Excluded from the analysis were 19 dogs (20 instances of hospitalization): because of incomplete medical records (*n* = 9), because emergency antiepileptic treatment was not standardized (*n* = 6), and because the management of convulsive activity was not directly supervised by a board-certified neurologist or a neurology resident (*n* = 5). The final sample was 37 dogs (50 instances of hospitalization): 24/37 (65%) were male (23 intact, 1 neutered) and 13/37 (35%) were female (8 intact, 5 neutered). The median weight was 23 kg (IQR 16–28.8). Most dogs were mixed breed (11/37) or border collie (6/37), German Shepherd (3/37), and 2 dogs each French Bulldog, English Bulldog, and American Staffordshire terrier.

Idiopathic epilepsy was diagnosed in 21/37 (57%), (13/21 tier I and 8/21 tier II); structural epilepsy was diagnosed in 6/37 (16%) of which 4/6 confirmed and 2/6 suspected. A metabolic or toxic cause of seizure activity was recorded in 7/37 (19%): suspected intoxication in 3/7, hypoglycemia in 2/7, hepatic encephalopathy in 1/7, electrolyte abnormalities in 1/7. The cause of seizures could not be established due to lack of diagnostic investigation in 3/37 (8%).

A total of 38/50 (76%) hospitalizations were noted for CS and 12/50 (24%) for SE. All episodes were represented by generalized motor seizures. The number of seizures before hospitalization for CS was available for 21/38 (55%) cases; the median number of episodes was 6 (IQR 4.5–10). The duration of SE before hospitalization was available for only 4/12 (33%) patients, the range of duration was from 10 to 150 min.

A history of previous seizures was recorded in 41/50 (82%) cases, 37/41 of which (90%) had been receiving AED treatment at presentation: 18/37 (49%) were receiving 1 AED [phenobarbital (PB)]; 13/37 (35%) were receiving 2 AEDs [10/13 PB and LEV, 3/13 PB and potassium bromide (KBr)]; 3/37 (8%) were receiving 3 AEDs (2/3 PB, LEV, Imepitoin, 1/3 PB, LEV, and KBr), and 1/37 (3%) was receiving 4 medications (PB, LEV, KBr, gabapentin). In 2/37 patients (5%), the long-term AED medication(s) were not recorded.

CRI of DZP or PPF was administered in 29/50 (58%) and in 21/50 (42%) instances of hospitalization, respectively. The median duration of CRI of DZP or PPF was 24 h (range, 12–28 h for DZP and 12–33 h for PPF). In all patients O2 supplementation was provided by nasal cannula and adequate blood oxygen saturation was assured by periodical evaluations, but no mechanical ventilation during CRI was required in any of these patients.

In 30/50 (60%) instances of hospitalization, the patient responded well to CRI with cessation of seizure activity, no recurrence in the 24 h after discontinuation of CRI through to hospital discharge, whereas a poor outcome was recorded for 20/50 (40%) cases (DZP CRI in 12/50 and PPF CRI in 8/50). Spontaneous death after sudden cardiac arrest while receiving CRI was recorded in 3/20 (15%) dogs, while 10/20 (50%) were humanely euthanized. Euthanasia was performed in 7/10 patients due to seizure recurrence despite treatment and in 3/10 patients due to catastrophic clinical consequences of seizure activity in 3/10. In particular, one dog developed ad ingestis pneumonia, while the other 2 manifested cardiac arrhythmias, acute renal failure, rhabdomyolysis and hemorrhagic diarrhea. In those patients, results of further tests such as blood gas and electrolyte analysis, hematological investigations, and FAST ultrasound of thorax and abdomen, along with thoracic radiographs concurred to the decision of euthanasia. Further medications to control seizure activity were administered in 7/20 (35%): 5/7 (51%) patients receiving CRI of DZP were switched to CRI of PPF. In 2/7 (29%) patients receiving CRI of PPF, a first attempt at weaning off was made then CRI was restarted due to recurrence of seizure activity within 24 h after discontinuation.

Table 1 presents the characteristics of the two groups and a comparison of the study variables and outcomes. There were no statistically significant differences in any of the variables studied.

**Table 1:** Outcome after CRI of DZP or PPF in dogs presenting with CS or SE: details of study groups and statistical analysis.

**Characteristic**	**Total hospitalisations *N* = 50 (%)**	**Good outcome *N* = 30 (%)**	**Poor outcome *N* = 20 (%)**	***p*-value**
**Constant rate infusion**				
DZP	29 (58)	17 (57)	12 (60)	
PPF	21 (42)	13 (43)	8 (40)	
				*P* = 0.76
**Sex**				
Males	35 (70)	19 (64)	16 (80)	
Males castrated	1 (2)	1 (3)	0 (0)	
Female	8 (16)	6 (20)	2 (10)	
Female neutered	6 (12)	4 (13)	2 (10)	
				*P* = 0.71
**Presentation**				
CS	38 (76)	22 (73)	16 (80)	
SE	12 (24)	8 (27)	4 (20)	
				*P* = 1
**Etiology**				
Idiopathic	34 (68)	22 (73)	12 (60)	
		13/22 DZP	8/12 DZP	
		2/22 PPF	4/12 PPF	
Structural	6 (12)	3 (10)	3 (15)	
		2/3 DZP	0/3 DZP	
		1/3 PPF	3/3 PPF	
Reactive	7 (14)	5 (17)	2 (10)	
		2/5 DZP	1/2 DZP	
		3/5 PPF	1/2 PPF	
Undefined	3 (6)	0	3 (15)	
			3/3 DZP	
			0/3 PPF	
				*P* = 0.12

Since a dose range of PPF was used during CRI, a statistical analysis was also performed to evaluate a potential dose effect of PPF CRI on outcome. The median dose of PPF CRI was similar in the two outcome groups [median dose PPF CRI good outcome: 0.150 (IQR 0.100–0.200); median dose PPF CRI bad outcome: 0.165 (IQR 0.100–0.200)] and no statistically significant differences were found (*p* = 0.97).

In patients with history of seizures, no statistically significant associations were found between outcome and presence or absence of chronic AED therapy, type of chronic AED(s) or number of chronic AED(s). Results are reported in Table 2.

**Table 2:** Outcome after CRI of DZP or PPF in dogs presenting with CS or SE in relation to antiepileptic treatment: details of study groups and statistical analysis.

**Previous AED (presence/absence)**	**Total population *N* = 41 (%)**	**Good outcome *N* = 24 (%)**	**Poor outcome *N* = 17 (%)**	***p*-value**
Yes	37 (90)	23 (96)	14 (82)	
No	4 (10)	1 (4)	3 (12)	
				*P* = 0.28
**Previous AED (number)**	**Total population** ***N*** = **39 (%)**	**Good outcome** ***N*** = **23 (%)**	**Poor outcome** ***N*** = **16 (%)**	***p*** **-value**
0 AED	4 (10)	1 (4)	3 (19)	
1 AED	18 (47)	11 (48)	7 (43)	
2 AED	13 (33)	10 (44)	3 (19)	
3 and 4 AED	4 (10)	1 (4)	3 (19)	
				*P* = 0.15
**Previous AED (type)**	**Total population** ***N*** = **31 (%)**	**Good outcome** ***N*** = **21 (%)**	**Poor outcome** ***N*** = **10 (%)**	***p*** **-value**
PB	18 (58)	11 (52)	7 (70)	
PB + LEV	10 (32)	8 (38)	2 (20)	
PB + KBr	3 (10)	2 (10)	1 (10)	
				*P* = 0.73

The median duration of hospitalization for patients with a good outcome overall was 36 h (IQR 24–48): the median duration was 24 h (IQR 24–36) for the dogs that received CRI of DZP and 36 h (IQR 12–60) for those that received CRI of PPF. Different degrees of ataxia and sedation were the only side effects recorded.

## Discussion

To our best knowledge, this is the first retrospective study to describe a fairly large sample of dogs treated with CRI of DZP or PPF because of lack of response to standard emergency treatment for CS or SE. Response to treatment was good overall, with complete suppression of seizure activity within 24 h after discontinuation of CRI through to hospital discharge.

A higher percentage of successful outcome was reported in case of CRI of DZP compared to PPF. A plausible explanation is the proven synergy in anti-seizure effect between LEV and DZP (9, 10) since all patients received one administration of LEV as part of the standardized baseline antiepileptic protocol. In contrast, it may be argued that since the present study lacks randomization, CRI of PPF was administered in more severe clinical cases with a worse prognosis. Caution is therefore warranted when drawing conclusions on the efficacy of CRI of DZP vs. PPF. Further studies are needed to elucidate the differences between the two types of AED.

Though consensus on the treatment of CS and SE is lacking, general guidelines suggest the administration of CRI with benzodiazepines or PPF if first-line therapy with benzodiazepines IV boluses combined with initiation of a maintenance AED such as Pb or LEV fails (4, 6, 11, 12). Despite these indications, and except for textbook information on dosages (6), however, to our best knowledge there is only one published report describing in detail the administration of CRI of midazolam in dogs (7) and very few studies on such treatments in clinical practice (1, 13–15).

Bateman and colleagues reported on CRI of DZP in 66.8% of hospital admissions in 156 cases of CS and SE, with a mean duration of 22.3 ± 16.1 h, but without further indications about medication dosage or efficacy (1), thus precluding comparison with the present study.

In their recent study, Bray and colleagues reported on the administration of CRI of midazolam in dogs referred for CS or SE. The mean duration of CRI was 25 h (range, 2–96 h). Complete control of seizure activity was defined as the absence of further seizures between CRI initiation or escalation and hospital discharge. Seizure activity was defined as largely controlled in case of a single, isolated seizure after CRI initiation without the need for dosage adjustment. In both cases, seizure control was considered successful. CRI of midazolam was associated with successful outcome in 85.4% cases (7). In contrast, we noted a lower response rate, also when only patients administered CRI of DZP were considered. The discrepancy may be related to the difference in intrinsic efficacy of the two benzodiazepines. In human medicine only one prospective study published to date has compared the efficacy of CRI of midazolam vs. DZP in controlling seizure activity during refractory SE in children. There were no statistically significant differences in outcome between the two groups (16). In veterinary medicine no clinical trials have compared benzodiazepines in the treatment of refractory SE. One study compared the efficacy of intranasal midazolam vs. rectal DZP in the emergency management of SE in dogs. The clinical trial involved 35 dogs with different seizure etiologies and concluded that intranasal midazolam was more efficacious than rectal DZP in controlling SE (17).

Based on this scant and contrasting information, the low responder rate we noted compared to Bray and colleagues may be explained by the greater efficacy of midazolam compared to DZP. However, our definition of successful outcome was stricter compared to the one Bray and colleagues applied, so it is possible that this difference in definition could have accounted for the overall lower success rate we found. Also, a direct comparison between the two studies is further complicated by the lack of a standardized baseline treatment protocol in patients receiving CRI of midazolam.

CRI of PPF for the treatment of refractory SE has been widely described in human medicine (18), whereas no veterinary studies have investigated this type of treatment. Steffen and Grasmueck reported on the use of boluses of PPF at a variable dosage of 2 to 8 mg/kg in combination with other AEDs to control refractory seizures of various origin (except metabolic-toxic) in dogs and cats, however, CRI was not administered (19). Later, CRI of PPF at different dosages and duration was reported to be part of the treatment plan for seizures following portosystemic shunt ligation in a small population of dogs and cats (13–15). To date, no studies have involved a larger population of patients with epilepsy of different etiology. In our study, the paucity of dogs with seizure activity of metabolic-toxic origin precludes comparison and conclusion regarding the administration of CRI of PPF in this kind of patients.

CRI of DZP and of PPF were well tolerated; the only side effects were sedation and ataxia. As reported in a previous study, these side effects cannot be separated from the consequences of seizure activity or the concomitant administration of other AEDs (7).

There is no standardized duration of CRI for epilepsy in veterinary medicine. The only indications that can be found in the literature regard CRI of benzodiazepine: the dosage rate should be reduced by 50% every 6 h for at least two reductions before discontinuation, while the duration of CRI of PPF should be at least 6 h (6, 12). The median duration of CRI of DZP in the present study is similar to that reported by Bray and colleagues for CRI of midazolam and by Bateman and colleagues for CRI of DZP (1, 7), while longer duration of up to 138 h has been reported for CRI of PPF (13–15).

In human medicine the guidelines for managing refractory seizure activity recommend induction of therapeutic coma for 24–48 h (20–22). However, debate revolves around whether the induction of therapeutic coma *per se* and its duration may be associated with increased mortality, poor functional outcome, increased risk of complications, and prolonged hospitalization (23–29). In their recent study, Mulhofer and colleagues reported that a shorter, yet deeper therapeutic coma may be more effective and safer than the current recommendation of 24–48 h of duration (28). In our study the duration of CRI was quite similar for both DZP and PPF, so it is impossible to speculate about the possible effects of CRI duration on outcome and complications. Prospective studies to evaluate this aspect are underway.

Our results show no association between clinical presentation (CS vs. SE) and treatment outcome. Although SE has been conventionally defined as a worse clinical neurological condition compared to CS, only one study to date has evaluated the potential risk factors for mortality in dogs with CS or SE, in which the clinical presentation was a variable of interest. The study found no association between SE and mortality *per se* (30). Our data seem to share this finding.

The duration of convulsive activity before treatment in case of SE was available only for a small percentage of patients, precluding the possibility to assess the influence of this parameter on the outcome and the treatment strategy itself. It is in fact well-known how during prologued seizure activity synaptic gamma amino-butyric acid (GABA)_a_ receptors undergo a progressive maladaptive internalization, causing loss of benzodiazepine efficacy (31). At the same time, glutamatergic excitation increases due to the upregulation of N-methyl-D-aspartate (NMDA) receptors (32). Based on these theories, experimental studies on rats have been conducted in order to evaluate the benefit of an early polytherapy approach, combining a GABA_a_ receptors agonist (diazepam or midazolam) and a NMDA receptor antagonist (ketamine) in treating SE with good results (33–35). Only one consistent retrospective study has been published on the utilization of ketamine in dogs affected by SE, refractory SE and CS. In those 15 cases reported, ketamine was administered at a dosage of 5 mg/kg IV boluses and resulted effective in 100% of cases of refractory SE, supporting the results obtained in experimental settings (36). In the present study, the treatment protocol did not include an NMDA receptor antagonist, therefore it is possible, but not confirmable, that some of the cases of poor outcome could be related to a reduction of efficacy of benzodiazepines in case of prolonged seizure activity, overexpression of NMDA receptors or both.

In the present study, a high percentage of dogs had a diagnosis of idiopathic epilepsy. This finding contrasts with other studies that reported a metabolic-toxic disease as the main cause of SE in the emergency settings (3, 37). However, other literature sources seem to contest this information, reporting 40.3–59% of dogs affected by IE displaying episodes of SE (2, 38). Furthermore, it must be noted that in our retrospective study both CS and SE were considered. It is well-known from previous studies that certain dog breeds, including the German Shepherd Dog and Border Collie, are predisposed to the development of CS in case of IE (39). These two breeds were the most represented in our population after mixbreed dogs (whom some individuals could also potentially have been genetically associated with German shepherds or border collies). This aspect may therefore also concur explaining the high percentage of dogs with IE presenting with CS.

A structural etiology of seizures has been associated with a worse prognosis (3, 40, 41). We noted no association between structural epilepsy and poor outcome in dogs administered CRI. The number of patients with structural epilepsy was very small in both groups, which may be the reason for the lack of a statistically significant difference.

In the present study, further statistical analyses were performed to verify the effect of previous AEDs therapy on outcome in our population. It is in fact well-known that PB is a potent cytochrome P450 inducer, whose enzymes cause a reduction of action of several medications, including PB itself (42). According to the results of the study, previous administration of PB seems to have no effect on outcome in our population of dogs. This analysis confirms in a way the results of a previous study assessing risk factors for mortality in dogs affected by CS and SE. According to that retrospective study, epileptic patients not receiving any AEDs at the time of presentation were 16 times more likely to display a poor outcome (30).

The duration of hospitalization in the present study cannot be compared with previous ones. It was not reported in a retrospective study of CRI of midazolam (7), while Bateman and colleagues reported a mean and median duration of hospitalization for the entire population of dogs, not only those receiving CRI, so it is impossible to make a direct comparison between ours and their study (1).

Owing to the retrospective study design, there are limitations on the information we were able to retrieve: medical record entry inaccuracies and non-uniform recording may have reduced the amount of useful data for our analysis. As previously stated, even though the emergency treatment protocol was standardized for all patients included in the study, the choice between DZP or PPF CRI was made without randomization, therefore conclusions on efficacy must be drawn with caution. Furthermore, the therapeutic efficacy of the CRI was only defined clinically by the cessation of visible seizure activity, and the lack of an EEG monitoring does not allow to confirm this clinical observation with quantifiable endpoints such as EEG burst suppression. These limitations notwithstanding, the present study is the first to document administration of CRI of DZP or PPF in a large sample of dogs with epilepsy. The medications appeared to be tolerated without major side effects and helped control seizure activity in most patients regardless of seizure etiology. Further studies are needed to evaluate the effects of CRI duration on outcome and complications.

## Data availability statement

The raw data supporting the conclusions of this article will be made available by the authors, without undue reservation.

## Ethics statement

Ethical review and approval was not required for the animal study because Ethical review and approval was not required for the animal study because of the retrospective nature of the study. Written informed consent was obtained from the owners for the participation of their animals in this study.

## Author contributions

GC and AD'A study design, data collection, statistical analysis, interpretation of results and generation of the manuscript, critical revision and final approval of version to be published. SF data collection, statistical analysis, interpretation of results and generation of the manuscript, critical revision, and final approval of version to be published. CB statistical analysis, interpretation of results, critical revision, and final approval of version to be published. GM, GB, CC, LM, and EA data collection, critical revision, and final approval of version to be published. All authors contributed to the article and approved the submitted version.

## Conflict of interest

The authors declare that the research was conducted in the absence of any commercial or financial relationships that could be construed as a potential conflict of interest.

## Publisher's note

All claims expressed in this article are solely those of the authors and do not necessarily represent those of their affiliated organizations, or those of the publisher, the editors and the reviewers. Any product that may be evaluated in this article, or claim that may be made by its manufacturer, is not guaranteed or endorsed by the publisher.

## References

[1] Bateman SW Parent JM. Clinical findings, treatment, and outcome of dogs with status epilepticus or cluster seizures: 156 cases (1990–1995). J Am Vet Med Assoc. (1999) 215:1463–8. 10579043

[2] Saito M Munana KR Sharp NJH Olby NJ. Risk factors for development of status epilepticus in dogs with idiopathic epilepsy and effects of status epilepticus on outcome and survival time: 32 cases (1990–1996). J Am Vet Med Assoc. (2001) 219:618–23. 10.2460/javma.2001.219.618 11549089

[3] Zimmermann R Hülsmeyer V-I Sauter-Louis C Fischer A. Status epilepticus and epileptic seizures in dogs. J Vet Intern Med. (2009) 23:970–6. 10.1111/j.1939-1676.2009.0368.x 19737178

[4] Patterson ENE. Status epilepticus and cluster seizures. Vet Clin North Am Small Anim Pract. (2014) 44:1103–12. 10.1016/j.cvsm.2014.07.007 25245182

[5] Charalambous M Volk HA Van Ham L Bhatti SFM. First-line management of canine status epilepticus at home and in hospital-opportunities and limitations of the various administration routes of benzodiazepines. BMC Vet Res. (2021) 17:1–19. 10.1186/s12917-021-02805-0 33663513 PMC7934266

[6] Platt SR. Pathophysiology and management of status epilepticus. In:De Risio L Platt SR, editors. Canine and Feline Epilepsy. Wallingford: CABI (2014), 519–536. 10.1079/9781780641096.0519

[7] Bray KY Mariani CL Early PJ Muñana KR Olby NJ. Continuous rate infusion of midazolam as emergent treatment for seizures in dogs. J Vet Intern Med. (2021) 35:388–96. 10.1111/jvim.15993 33325618 PMC7848341

[8] Berendt M Farquhar RJ Mandigers PJ Pakozdy A Bhatti SFM De Risio L. International veterinary epilepsy task force consensus report on epilepsy definition, classification and terminology in companion animals. BMC Vet Res. (2015) 11:1–11. 10.1186/s12917-015-0461-2 26316133 PMC4552272

[9] Mazarati AM Baldwin R Klitgaard H Matagne A Wasterlain CG. Anticonvulsant effects of levetiracetam and levetiracetam-diazepam combinations in experimental status epilepticus. Epilepsy Res. (2004) 58:167–74. 10.1016/j.eplepsyres.2004.02.002 15120747

[10] Modur PN Milteer WE Zhang S. Sequential intrarectal diazepam and intravenous levetiracetam in treating acute repetitive and prolonged seizures. Epilepsia. (2010) 51:1078–82. 10.1111/j.1528-1167.2009.02385.x 19845733 PMC3095965

[11] Platt SR. Pathophysiology and management of cluster seizures. In:De Risio L Platt SR, editors. Canine and Feline Epilepsy. Wallingford: CABI (2014), 503–518. 10.1079/9781780641096.0503

[12] Blades Golubovic S Rossmeisl JH. Status epilepticus in dogs and cats, part 2: treatment, monitoring, and prognosis. J Vet Emerg Crit Care. (2017) 27:288–300. 10.1111/vec.12604 28445601

[13] Heldmann E Holt DE Brockman DJ Brown DC Perkowski SZ. Use of propofol to manage seizure activity after surgical treatment of portosystemic shunts. J Small Anim Pract. (1999) 40:590–4. 10.1111/j.1748-5827.1999.tb03029.x 10664958

[14] Heidenreich DC Giordano P Kirby BM. Successful treatment of refractory seizures with phenobarbital, propofol, and medetomidine following congenital portosystemic shunt ligation in a dog. J Vet Emerg Crit Care. (2016) 26:831–6. 10.1111/vec.12431 26683894

[15] Gommeren K Claeys S de Rooster H Hamaide A Daminet S. Outcome from status epilepticus after portosystemic shunt attenuation in 3 dogs treated with propofol and phenobarbital. J Vet Emerg Crit Care. (2010) 20:346–51. 10.1111/j.1476-4431.2010.00537.x 20636988

[16] Singhi S Murthy A Singhi P Jayashree M. Continuous midazolam vs. diazepam infusion for refractory convulsive status epilepticus. J Child Neurol. (2002) 17:106–10. 10.1177/088307380201700203 11952069

[17] Charalambous M Bhatti SFM Van Ham L Platt S Jeffery ND Tipold A. Intranasal midazolam vs. rectal diazepam for the management of canine status epilepticus: a multicenter randomized parallel-group clinical trial. J Vet Intern Med. (2017) 31:1149–58. 10.1111/jvim.14734 28543780 PMC5508334

[18] Vossler DG Bainbridge JL Boggs JG Novotny EJ Loddenkemper T Faught E. Treatment of refractory convulsive status epilepticus: a comprehensive review by the american epilepsy society treatments committee. Epilepsy Curr. (2020) 20:245–64. 10.1177/1535759720928269 32822230 PMC7576920

[19] Steffen F Grasmueck S. Propofol for treatment of refractory seizures in dogs and a cat with intracranial disorders. J Small Anim Pract. (2000) 41:496–9. 10.1111/j.1748-5827.2000.tb03971.x 11105788

[20] Glauser T Shinnar S Gloss D Alldredge B Arya R Bainbridge J. Evidence-based guideline: treatment of convulsive status epilepticus in children and adults. Epilepsy Curr. (2016) 16:48–61. 10.5698/1535-7597-16.1.48 26900382 PMC4749120

[21] Brophy GM Bell R Claassen J Alldredge B Bleck TP Glauser T. Guidelines for the evaluation and management of status epilepticus. Neurocrit Care. (2012) 17:3–23. 10.1007/s12028-012-9695-z 22528274

[22] Meierkord H Boon P Engelsen B Göcke K Shorvon S Tinuper P. guideline on the management of status epilepticus in adults. Eur J Neurol. (2010) 17:348–55. 10.1111/j.1468-1331.2009.02917.x 20050893

[23] Marchi NA Novy J Faouzi M Stähli C Burnand B Rossetti AO. Status epilepticus: impact of therapeutic coma on outcome. Crit Care Med. (2015) 43:1003–9. 10.1097/CCM.0000000000000881 25654177

[24] Sutter R Marsch S Fuhr P Kaplan PW Rüegg S. Anesthetic drugs in status epilepticus: risk or rescue? Neurology. (2014) 82:656–64. 10.1212/WNL.0000000000000009 24319039 PMC3945664

[25] Sutter R Kaplan PW. Can anesthetic treatment worsen outcome in status epilepticus? Epilepsy Behav. (2015) 49:294–7. 10.1016/j.yebeh.2015.02.044 25819797

[26] Kowalski RG Ziai WC Rees RN Werner JK Kim G Goodwin H. Third-line antiepileptic therapy and outcome in status epilepticus: the impact of vasopressor use and prolonged mechanical ventilation. Crit Care Med. (2012) 40:2677–84. 10.1097/CCM.0b013e3182591ff1 22732291

[27] Sutter R De Marchis GM Semmlack S Fuhr P Rüegg S Marsch S. Anesthetics and outcome in status epilepticus: a matched two-center cohort study. CNS Drugs. (2016) 31:64–74. 10.1007/s40263-016-0389-5 27896706

[28] Muhlhofer WG Layfield S Lowenstein D Lin CP Johnson RD Saini S. Duration of therapeutic coma and outcome of refractory status epilepticus. Epilepsia. (2019) 60:921. 10.1111/epi.14706 30957219 PMC6571024

[29] Alvarez V Lee JW Westover MB Drislane FW Novy J Faouzi M. Therapeutic coma for status epilepticus. Neurology. (2016) 87:1650–9. 10.1212/WNL.0000000000003224 27664985 PMC5085074

[30] Cagnotti G Ferrini S Ala U Bellino C Corona C Dappiano E. Analysis of Early assessable risk factors for poor outcome in dogs with cluster seizures and status epilepticus. Front Vet Sci. (2020) 7:575551. 10.3389/fvets.2020.575551 33195572 PMC7581674

[31] Deeb TZ Maguire J Moss SJ. Possible alterations in GABA_A_ receptor signaling that underlie benzodiazepine-resistant seizures. Epilepsia. (2012) 53:79–88. 10.1111/epi.12037 23216581 PMC4402207

[32] Naylor DE Liu H Niquet J Wasterlain CG. Rapid surface accumulation of NMDA receptors increases glutamatergic excitation during status epilepticus. Neurobiol Dis. (2013) 54:225–238. 10.1016/j.nbd.2012.12.015 23313318 PMC5444936

[33] Martin BS Kapur J. A combination of ketamine and diazepam synergistically controls refractory status epilepticus induced by cholinergic stimulation. Epilepsia. (2008) 49:248–55. 10.1111/j.1528-1167.2007.01384.x 17941842 PMC2844443

[34] Niquet J Baldwin R Norman K Suchomelova L Lumley L Wasterlain CG. Midazolam–ketamine dual therapy stops cholinergic status epilepticus and reduces Morris water maze deficits. Epilepsia. (2016) 57:1406–15. 10.1111/epi.13480 27500978 PMC5012923

[35] Niquet J Baldwin R Norman K Suchomelova L Lumley L Wasterlain CG. Simultaneous triple therapy for the treatment of status epilepticus. Neurobiol Dis. (2017) 104:41–9. 10.1016/j.nbd.2017.04.019 28461248 PMC5504687

[36] Roynard P Bilderback A Dewey CW. Intravenous Ketamine Bolus(es) for the treatment of status epilepticus, refractory status epilepticus, and cluster seizures: a retrospective study of 15 dogs. Front Vet Sci. (2021) 8:547279. 10.3389/fvets.2021.547279 33681317 PMC7925624

[37] Zimmermann R Steinberg TA Raith K Hulsmeyer V Fischer A. Canine status epilepticus due to acute intoxication. Tierarztl Prax Ausgabe K Kleintiere Heimtiere. (2010) 38:285–94. 10.1055/s-0038-1622862 22215313

[38] Fentem R de Stefani A Quintana RG Alcoverro E Jones GMC Amengual-Batle P. Risk factors associated with short-term mortality and recurrence of status epilepticus in dogs. J Vet Intern Med. (2022) 36:656–62. 10.1111/jvim.16353 34994484 PMC8965210

[39] Hülsmeyer VI Fischer A Mandigers PJJ DeRisio L Berendt M Rusbridge C. International veterinary epilepsy task force's current understanding of idiopathic epilepsy of genetic or suspected genetic origin in purebred dogs. BMC Vet Res. (2015) 11:1–28. 10.1186/s12917-015-0463-0 26316206 PMC4552344

[40] Fredsø N Koch BCC Toft N Berendt M. Risk factors for survival in a university hospital population of dogs with epilepsy. J Vet Intern Med. (2014) 28:1782–8. 10.1111/jvim.12443 25252168 PMC4895623

[41] Hardy BT Patterson EE Cloyd JM Hardy RM Leppik IE. Double-masked, placebo-controlled study of intravenous levetiracetam for the treatment of status epilepticus and acute repetitive seizures in dogs. J Vet Intern Med. (2012) 26:334–40. 10.1111/j.1939-1676.2011.00868.x 22295898

[42] Brodie MJ Mintzer S Pack AM Gidal BE Vecht CJ Schmidt D. Enzyme induction with antiepileptic drugs: cause for concern? Epilepsia. (2013) 54:11–27. 10.1111/j.1528-1167.2012.03671.x 23016553

